# Detection of meat from horse, donkey and their hybrids (mule/hinny) by duplex real-time fluorescent PCR

**DOI:** 10.1371/journal.pone.0237077

**Published:** 2020-12-29

**Authors:** Dan Wang, Liping Wang, Chenyu Xue, Yuebei Han, Hejing Li, Jianqiang Geng, Jiang Jie

**Affiliations:** Biology Lab, Beijing Municipal Center for Food Safety Monitoring and Risk Assessment, Beijing, China; University of Helsinki, FINLAND

## Abstract

Meat adulteration is currently a common practice worldwide. In China, adulteration of donkey meat products with the similar species (horse and mule/hinny) meat and mislabeling are becoming widespread concerns. In this study, a sensitive and species-specific duplex real-time PCR assay based on the simultaneous amplification of fragments of the creatine kinase muscle gene family, was developed and optimized for the identification of horse, donkey and mule /hinny species in raw and heat-processed meat products. Duplex real-time PCR results showed different fluorescence amplification curves for horse and donkey. Both kinds of fluorescence amplification curves appeared simultaneously for mule/hinny. The limit of detection (LOD) of the method was up to 0.01 ng /μL. The method and strategy developed in this study could be applied to detect the presence of adulterants from horse and mule /hinny meat in raw donkey meat and meat products.

## Introduction

Donkey-related meat products have received attention due to their high nutritional value and unique taste in China. Due to donkey resource scarcity, long growth cycle, and gradually increasing market demand, the price of donkey meat is continuously rising. Donkey meat with other low-cost animal meat, especially from similar species, including horse and mule or hinny, is common in markets and in restaurants [1, 2]. Because horse, donkey and mule/hinny are equine animals, it is difficult to accurately identify the source of meat through their muscle structure. The fraudulent substitution or undeclared adulteration of meat species in comminuted and highly processed meat products could bring potential risks to people who have allergies to certain types of meat proteins. Furthermore, consuming food containing donkey and horse sources is haram for Muslims. Therefore, it is particularly important and urgent to establish a fast, accurate and reliable method for identifying adulterated donkey meat.

The methods currently applied to detect meat adulteration rely on either protein or DNA analysis. Protein-based techniques have a number of limitations due to the denaturation of protein during thermal processing. DNA is stable even with processing or heat treatment. DNA-based techniques include PCR [3], real-time PCR [4, 5], digital PCR [6], restriction fragment length polymorphism(RFLP) [7], DNA hybridization [8], and DNA barcoding [9]. In particular, the real-time PCR method has been widely applied for meat adulteration identification. These assays are rapid and can be used for routine high-throughput screening of multiple samples. Additionally, real-time PCR reduces the potential of contamination of the PCR mixture as the reaction tubes remain closed throughout the assay. Research papers from different countries, including the USA [10], Germany [11], Switzerland [12, 13], Portugal [14], and Malaysia [15], for the detection of meat origin and adulteration with this method have been published.

Animal mitochondrial DNA was the research target in these technical methods because of its high sensitivity and specificity. Moreover, these detection techniques based on DNA overcome the limitations of the animal meat fiber structure, have a much higher objectivity and accuracy, and are suitable for the classification and identification of species. Research papers related to identification of donkey and horse meat have also been published [16–18]. However, mitochondrial DNA is not applicable for the detection of hybrid animals because the mode of inheritance of mitochondria is generally thought to be strictly maternal [19, 20]. Mules are the offspring of jacks and mares, and hinnies are the offspring of stallions and jennies. The close relationship makes the mitochondrial DNA of these animals very similar Therefore, nuclear DNA is the optimal choice for the identification of hybrid animal species present in meat products [21].

Mule and hinny are hybrids of horse and donkey. They have both donkey and horse nuclear genes in their cytoblasts, there are 32 chromosomes in horses and 31 chromosomes in donkey. Few articles or standard identification methods which aim at nuclear genes by fluorescent quantitative PCR methods for the detection ingredient of mule or hinny in raw meat have been published.

Hence, in this study, a duplex PCR assay with two species-specific probes for donkey and horse was developed and validated, and distinguish donkey from horse and mule/hinny by showing different fluorescence amplification curves. The goals of this study were to identify fraudulently imitated donkey meat from mule/hinny and to provide a precise method to aid law enforcement agencies in the control of food adulteration.

## Materials and methods

### Test material preparation

Authentic frozen lean meat of multiple species of animals used as reference material was obtained from our lab and saved at -20°C. These species included cow (*Bos taurus*), sheep (*Ovisaries*), goat (*Capra hircus*), donkey (*Equus asinus*), horse (*Equus caballus*), chicken (*Gallus gallus*), duck (*Anas platyrhynchos*), goose (*Anse ranser*), turkey (*Meleagris gallopavo*), pig (*Sus scrofa*), quail (*Coturnix coturnix*), camel (*Camelus dromedarius*), dog (*Canis lupus familiaris*), ferret (*Mustela putorius furo*), rabbit (*Oryctolagus cuniculus*), pigeon (*Columba livia*), mouse (*Mus musculus*), rat (*Rattusnorvegicus*), deer (*Cervus nippon*) and fox (*Vulpes*). Mule/hinny blood samples were collected from farmers who raised mules/hinnies for farm work. All the market samples were purchased from supermarkets, inns, farmers' markets and online stores. All samples (whether raw or heat-processed meat) were minced with meat grinders, placed in clean plastic bags and stored at -20°C until DNA extraction.

### DNA extraction

Total genomic DNA was extracted from 200 mg of each meat sample by using a TIANamp Genomic DNA Kit (Tiangen, China) according to the supplier’s manual. Following DNA extraction, the purity and concentration of the DNA solutions were measured by UV photometry with a NanoDrop 1000 Spectrophotometer (Thermo, USA). Extracted DNA samples(100 μL) were placed in a 1.5 mL microcentrifuge and stored at -20°C.

### Primers and probes

**Fig 1:**
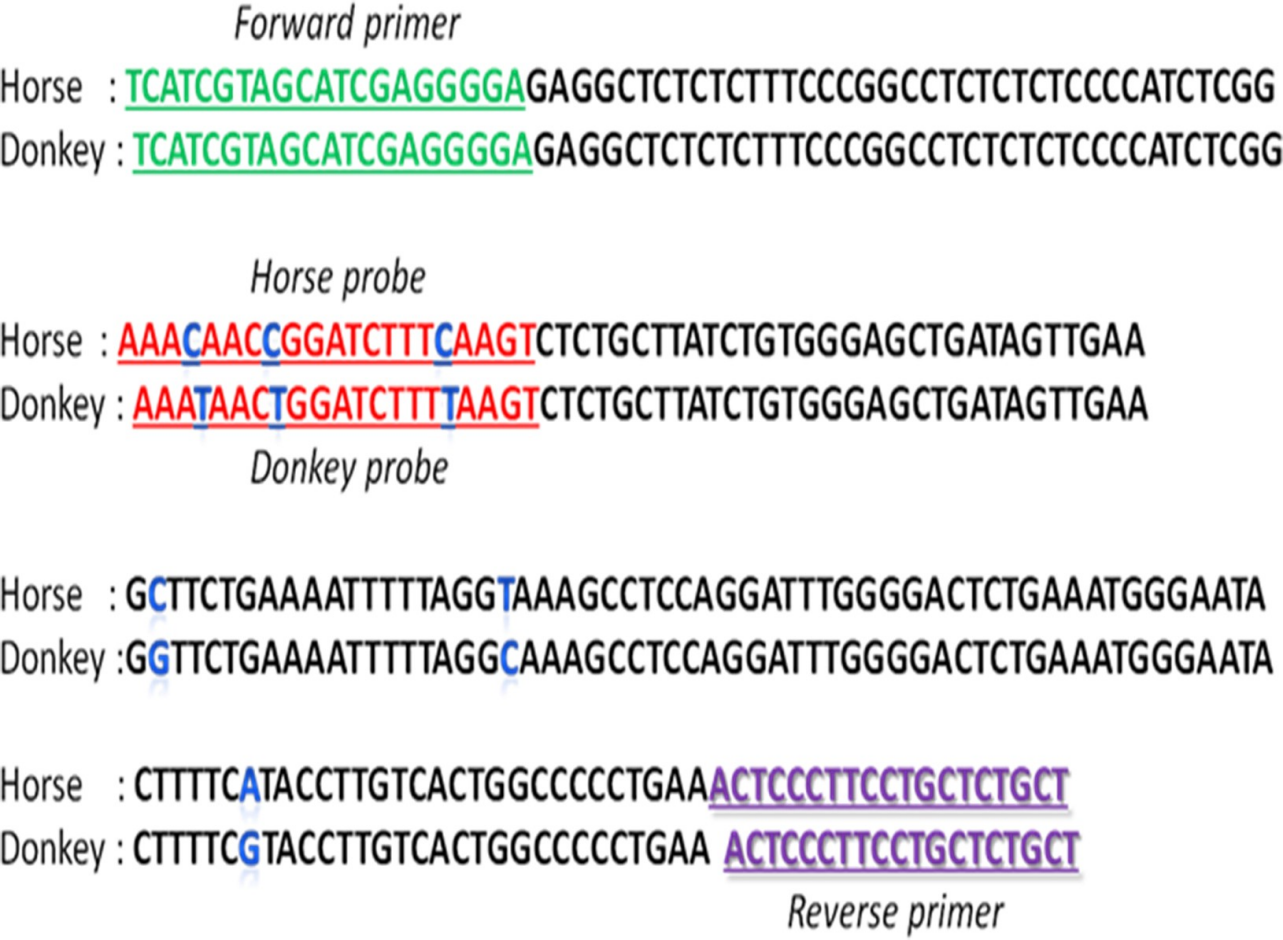
MCK sequences of horse and donkey used in duplex real-time PCR. Green characters: universal forward primer sequence; purple characters: universal reverse primer sequence; red characters: probe sequence; blue character: differing bases between horse and donkey MCK gene sequences.

The housekeeping gene, which encodes creatine kinase muscle (MCK), was selected as the target detection sequence. MCK sequences from donkeys and horses were searched against representative species using BLAST. Primers and probes were designed using Primer Premier 5.0, and target sequences were aligned using DNAMAN. The length of the amplified fragment was 217 bp (Fig 1) Universal primers were designed for horse and donkey (Table 1).

**Table 1:** Primers and probes used for duplex real-time PCR assays.

Primer/Probe	Sequence/labeling	NCBI Reference Sequence
HD-F	5’-TCATCGTAGCATCGAGGGGA-3’	HM151344.1
HD-R	5’-AGCAGAGCAGGAAGGGAGT-3’	HQ336263.1
Horse-P	5’-(FAM)AAACAACCGGATCTTTCAAGT(MGB) -3’	HM151344.1
Donkey-P	5’-(VIC)AAATAACTGGATCTTTTAAGT(MGB) -3’	HQ336263.1

### Duplex real-time PCR quantification approaches

The 20 μL PCR reaction mixture consisted of 0.6 μL of each primer (10 μM), 0.3 μL of horse-probe (10 μM), 0.5 μL of donkey-probe (10 μM),10 μL of 2xSuperReal PreMix (Tiangen, China), 1 μL of template DNA (20 ng~40 ng), and 7 μL of nuclease-free water (Tiangen, China). PCR was performed on an Applied Biosystems 12K Real-time system (Applied Biosystems, USA) according to the following cycling protocol: an initial step of 3 min at 95°C; followed by 40 cycles of 3 s at 95°C and 32 s at 62°C.

### Specificity

To evaluate the species specificity in the assay, DNA from different animal species was used as templates for duplex real-time PCR. These animal species are listed in the test material preparation section.

### Detection sensitivity and repeatability

To test the detection sensitivity of the duplex real-time PCR in detecting meat from these three species, DNA-extracts of each target species (100 ng/μL DNA) were diluted to 0.01 ng/μL by a 10-fold series dilution in logarithmic steps with 0.2x TE buffer. Three replicates of PCR tests and three independent experiments were run for each level. Five independent experiments were carried out to determine the repeatability of the method system. Repeatability (intra-run variation) for the assay was reported by the percent coefficient of variance (% CV) within the assays.

### Analysis of commercial samples

For testing the robustness and real-world performance, the developed method was used to test 158 samples, including 20 raw and 138 heat-processed meat samples that were purchased from inns and supermarkets and online stores. All samples were tested using the national standard method first. SN/T 3730.5–2013 was used to detect horse ingredient, and SN/T 3730.4–2013 was used to detect donkey ingredient.

## Results

### Target gene selection

The equine MCK gene is a housekeeping gene. In this study, the amplified fragment length was 217 bp. The sequence was identical to the sequence downloaded from GenBank. Only three different bases occurred in the amplified probe sequence fragment between horse and donkey DNA (Fig 1). The probes can completely distinguish these two kinds of DNA fragments.

### Specificity

**Fig 2:**
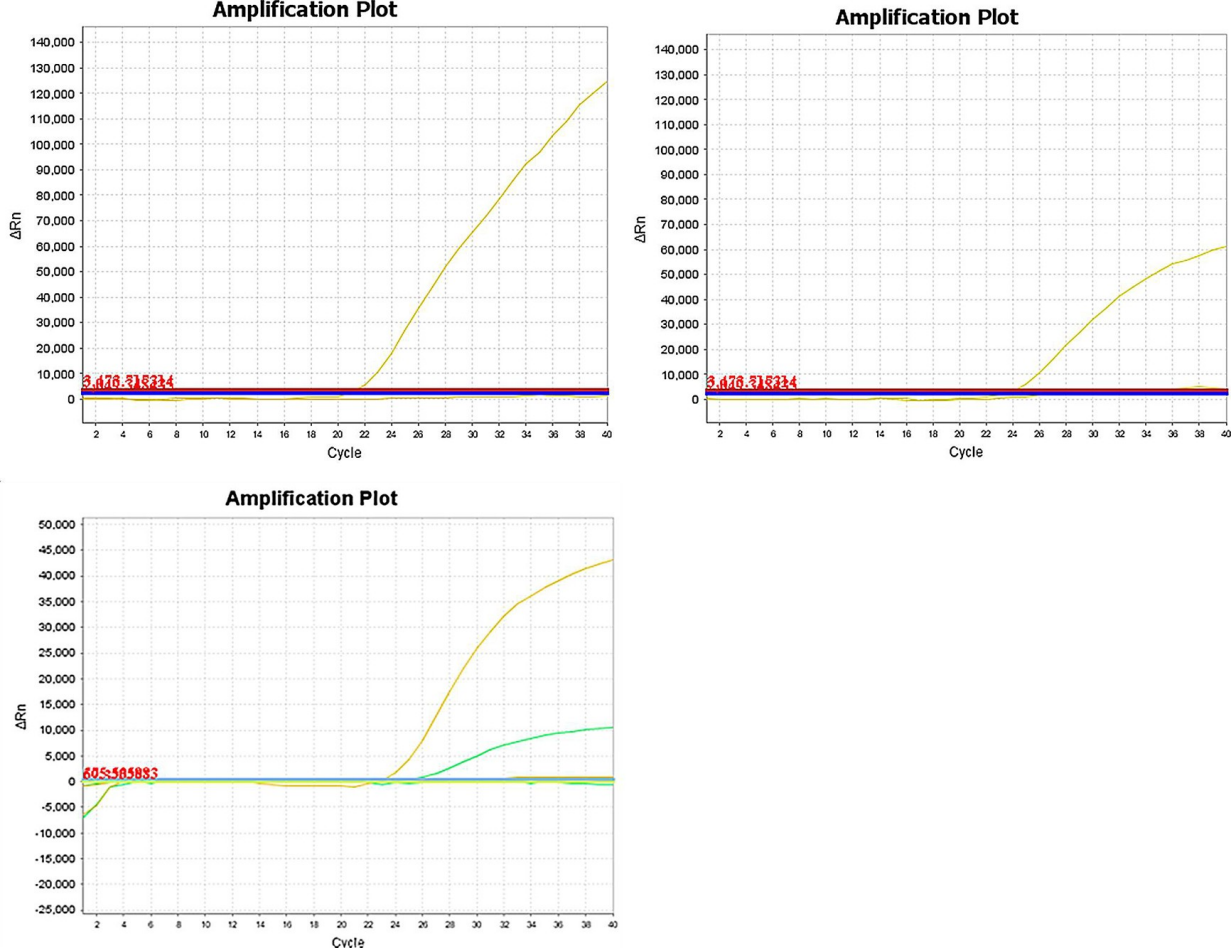
The specificity results of this duplex real-time PCR detection system. A. The specificity results of horse primers and probe. The fluorescence curve of the horse MCK gene. Real-time PCR amplification and detection of the horse MCK gene (probe labeled with the fluorescent dye FAM). B. The specificity results of donkey primers and probe. The fluorescence curve of the donkey MCK gene (probe labeled with fluorescent dye VIC). C. The specificity results of mule/hinny primers and probes. The fluorescence curve of the mule/hinny MCK genes. Real-time PCR amplification and detection of the mule/hinny MCK genes. The yellow fluorescence curve represents horse; the green fluorescence curve represents donkey.

The specificity of this duplex real-time PCR detection system was evaluated for the animal species listed above. Donkey and horse primer/probe combinations were evaluated in this real-time PCR detection system to assess the presence of cross-reactivity with other species. No cross-amplifications were observed, indicating that the primer/probe combinations used in this study could be used to specifically and reliably identify donkey, horse and mule/hinny materials in raw meat products (Fig 2).

### Sensitivity and repeatability

The LOD in this study was defined as the smallest concentration at which all sample replicates gave a positive qualitative result, indicating that the detection rate should be above 95% (Table 2). In three independent experiments, the duplex PCR detection method demonstrated good assay performance and high sensitivity. The LOD was determined to be as low as 0.01 ng/μL for donkey, horse and mule/hinny (S1 Fig). As a template, the DNA of mule/hinny is subjected to three reactions and three independent assays.

**Table 2:** Repeatability tests.

Mule/hinny Meat	Five Parallel Independent Tests					Mean Value	RSD	Coefficient of variation (%)
	1	2	3	4	5			
Horse (Ct)	24.31	24.33	24.58	24.45	24.63	24.46	0.23	0.82
Donkey(Ct)	26.13	26.42	26.34	26.56	26.27	26.34	0.32	1.14

Coefficient of variation: a measure of the dispersion of data points around the mean in a series.

The repeatability of the system was evaluated by variance. The results are shown in Table 2. The average values of Ct values of horse and donkey were 24.46 and 26.34, respectively. The standard deviations were 0.23 and 0.32, respectively. The assays were determined to have high repeatability with very low intra-run %CV, which were 0.82% and 1.14% respectively. The very low %CV (<5%) indicates that these assays have high repeatability. These results indicate that the detection limit of this test is accurate and stable and can be used for the detection of donkey, horse and mule/hinny.

### Detection of commercial samples

The duplex real-time PCR assay was then utilized for the detection of one hundred and fifty-eight market samples. Table 3 shows the results from all samples. In 20 raw meat samples, the test results coincide with the standard method. 4 samples were simultaneously detected horse and donkey in 78 heat–processed donkey meat samples and were hinnies. The remaining samples were derived from donkey. In addition, 9 samples were simultaneously detected horse and donkey in 60 heat–processed horse meat samples and were identified as mules. The remaining 51 samples were derived from horse. The results showed that the duplex real-time PCR assay is robust and could be successfully applied.

**Table 3:** Samples from the market were analyzed by duplex real-time PCR.

Type	Species	NO.	Standard method		Duplex real-time PCR			
			Horse	Donkey	Horse	Donkey	Mule/hinny	
Raw meat	Horse	10	10	0	10	0		0
Raw meat	Donkey	10	0	10	0	10		0
Heat-proceed product	Donkey	78	0	78	4	78		4
Heat-proceed product	Horse	60	60	0	60	9		9

## Discussion

Horse and donkey have close genetic backgrounds. They belong to the *Equine* family and have highly homologous sequences. Probe design is particularly important for distinguishing horse from donkey. Usual fluorescent probes have difficulty distinguishing highly similar template sequences and reduce the specificity of species origin [22, 23]. In this study, the length of the amplified sequence of the MCK gene was 217 bp. There are only three different bases in the common amplified sequences between horse and donkey, and they have been differentiated completely using the MGB probe. In both raw and heat-processed meat samples, their ingredients were identified by duplex real-time PCR. The heat-processed technique had no impact on DNA extraction from meat. This is due to the heat tolerance of DNA.

Mitochondrial DNA is widely used in species identification and is an effective genetic marker for lineage generation and migration in biological evolution [24, 25] Mitochondria are generally thought to be maternally inherited. These claims were supported by hybridization test results with donkey and horses, goat and sheep, and with progeny from different human mtDNAs [26]. Mitochondrial DNA has certain application limitation for identifying hybrid animal. For instance, the identification of mule or hinny meat cannot be detected by the standard detection methods in China, which take mitochondrial ATPase 6 as a target DNA sequence.

Nuclear DNA is stable in heredity and has a stable single copy number, which is more advantageous and prospective in animal species identification, especially in the identification of hybrid animals [27, 28]. Many studies for identifying animal species have been published using nuclear genomic DNA. Rehbein successfully established a PCR method for detecting fish-derived DNA based on nuclear genomic DNA [29]. Wu et al established a multiplex quantitative PCR method targeting nuclear genomic DNA for the detection of fox, dog and rabbit [30]. Satkoski et al have developed a species determination assay for common poultry species based on nuclear DNA [31]. These research results showed that nuclear DNA-based species determination is feasible. Xu et al found that the difference in mtDNA between horse and donkey was confirmed by a DNA endonuclease analysis [32]. The MCK gene is highly conserved and can be used to identify animal species with different genetic backgrounds. MCK is mainly expressed in different muscle cells and is highly expressed in cardiac and skeletal cells [33]. In this study, the primers and probes designed could distinguish meat originating from donkey, horse and mule/hinny in a duplex fluorescent PCR detection system. The quick and accurate detection of *Equus* species could reduce fraudulent replacement of donkey meat with horse and mule/hinny meat.

In conclusion, the utility of duplex real-time PCR for the detection of nuclear DNA retrieved from donkey, horse, and mule species in meat products has been demonstrated. The duplex real-time PCR showed different fluorescence amplification curves for horse and donkey. For mule/hinny, both horse and donkey fluorescence amplification curves appeared simultaneously. This new molecular technique was shown to be a highly specific, sensitive, and efficient tool for the determination of meat adulteration in both raw and heat-processed meat samples. This detection method is not applicable for the identification of mixed livestock meat. A detection method for mixed meat components would be studied further.

## Supporting information

S1 Fig Amplification plots from dilution series of genomic DNA from horse and donkey. Genomic DNA dilutions ranged from final concentration of 0.01 ng to 100 ng, in a total of 5 data points. The species were horse and donkey in A and B respectively.
(PDF)
