# Supplementary figures and images for: Detection of meat from horse, donkey and their hybrids (mule/hinny) by duplex real-time fluorescent PCR
Source: PLoS One. 2020 Dec 29;15(12):e0237077. doi: 10.1371/journal.pone.0237077 (PMC7771862; doi:10.1371/journal.pone.0237077)

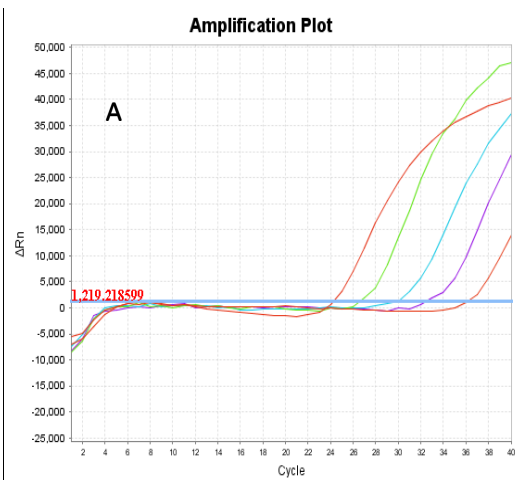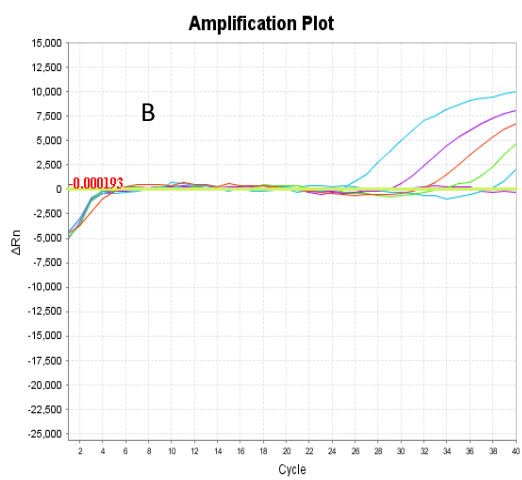

Supplement: S1 Fig — Genomic DNA dilutions ranged from final concentration of 0.01 ng to 100 ng, in a total of 5 data points. The species were horse and donkey in A and B respectively. (PDF) [file pone.0237077.s001.pdf]
